# Clinical significance of right ventricular–pulmonary arterial coupling in patients with tricuspid regurgitation before closure of atrial septal defect

**DOI:** 10.3389/fcvm.2022.896711

**Published:** 2022-11-14

**Authors:** Seon Hwa Lee, Yu Rim Shin, Dae-Young Kim, Jiwon Seo, Iksung Cho, Sak Lee, Jung Sun Kim, Geu-Ru Hong, Jong-Won Ha, Chi Young Shim

**Affiliations:** ^1^Division of Cardiology, Severance Cardiovascular Hospital, Yonsei University College of Medicine, Seoul, South Korea; ^2^Department of Cardiothoracic Surgery, Severance Cardiovascular Hospital, Yonsei University College of Medicine, Seoul, South Korea; ^3^Department of Cardiology, CHA Bundang Medical Center, CHA University, Seongnam, South Korea

**Keywords:** atrial septal defect, tricuspid regurgitation (TR), right ventricular-pulmonary artery coupling, echocardiography, old age

## Abstract

**Background:**

Functional tricuspid regurgitation (TR) usually decreases after atrial septal defect (ASD) closure; however, it may persist and cause heart failure that requires treatment. We aimed to investigate clinical and echocardiographic factors predicting persistent TR after ASD closure.

**Methods:**

Among 348 adults who underwent isolated ASD closure between January 2010 and September 2020, 91 (26.1%) patients with significant TR (at least moderate degree) before ASD closure were included. Persistent TR was defined as significant TR on echocardiography at 6 months to 1 year after ASD correction. We comprehensively analyzed the echocardiogram before ASD closure, including speckle-tracking imaging. Right ventricular (RV)–pulmonary arterial (PA) (RV–PA) coupling was assessed by the ratio of RV global longitudinal strain (RV GLS) and tricuspid annular S' velocity to PA systolic pressure (PASP).

**Results:**

Persistent TR was observed in 22 (24.2%) patients. Patients with persistent TR were significantly older and had larger TR jet areas and lower RV–PA coupling parameters than those without persistent TR. On multivariable regression, persistent TR was independently associated with age [odds ratio (OR) 1.07, 95% confidence interval (CI) 1.01–1.14, *p* = 0.030) and |RV GLS|/PASP (OR 0.001, 95% CI 0.00–0.017, *p* = 0.012). ROC curves analysis showed that |RV GLS|/PASP's best cut-off for persistent TR was 0.46 (cut-off 0.46, the area under the curve 0.789, *p* < 0.001).

**Conclusion:**

Persistent TR after ASD closure is not rare. Old age and RV–PA uncoupling could be associated with persistent TR after ASD closure. In older patients with abnormal RV–PA coupling, careful evaluation and concomitant or subsequent TR intervention may be considered.

## Introduction

Atrial septal defect (ASD) is a relatively common congenital heart disease in adults. The left-to-right shunt causes right ventricular (RV) volume overload and changes in pulmonary vasculature resulting in RV pressure overload (1). Finally, if ASD is not corrected, pulmonary hypertension can be induced. Functional tricuspid regurgitation (TR) is the result of RV volume overload and frequently occurs in adult patients with ASD (2, 3). Significant TR has been associated with cardiovascular morbidity and mortality (4, 5). Previous studies have addressed the effects of percutaneous and isolated surgical ASD closures in patients with significant TR (6, 7). In patients with ASD combined with significant TR, ASD closure is associated with a significant reduction in functional TR, over the long term (6, 8). However, a few studies reported that residual functional TR is common after device closure (2, 7). Understanding the regression of functional TR after ASD closure is important to determine the optimal therapeutic strategy for ASD [i.e., ASD occlusion alone or combined corrected tricuspid valve (TV) surgery].

Non-invasive estimation of RV–pulmonary arterial (PA) coupling, a load-independent measure of RV performance, using a ratio of RV systolic functional parameters and RV afterload (PA systolic pressure), has been validated as a prognostic marker in patients with PA hypertension, heart failure, and adult congenital heart disease (9, 10). In the present study, we aimed to examine the clinical and echocardiographic factors associated with persistent TR after ASD closure and whether RV–PA coupling and persistent TR after ASD closure are related.

## Methods

### Study population

**Figure 1:**
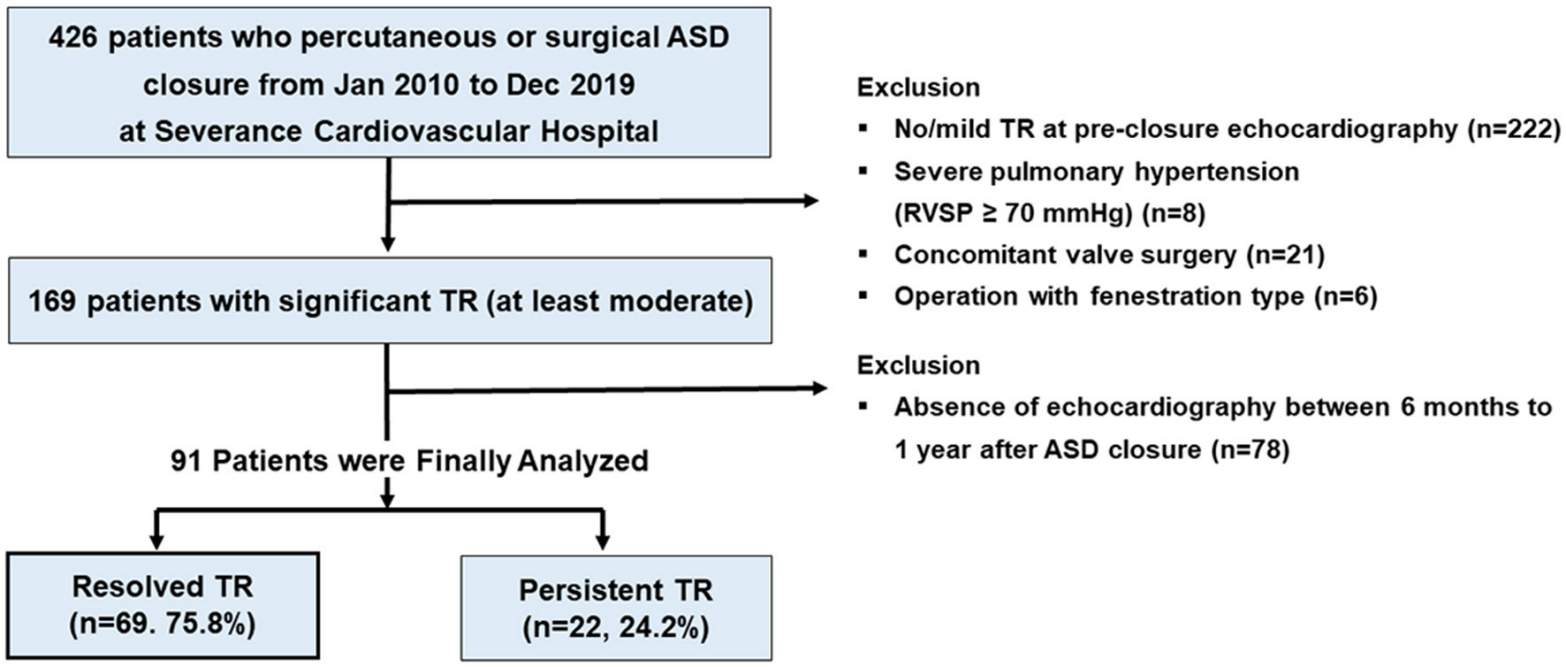
Graphical representation of the patient selection process.

This study included a total of 348 adults who underwent isolated ASD (primum and secondum) surgical or percutaneous closures without combined structural abnormality between January 2010 and September 2020 at a single tertiary hospital. In patients with ASD closure, echocardiography was routinely performed at baseline, before ASD closure, and at 6 months or 1 year after ASD closure. The exclusion criteria included the following: (1) no or mild TR at baseline echocardiography (*n* = 222); (2) concomitant valve surgery or congenital cardiac defects (*n* = 21); (3) severe pulmonary hypertension [right ventricular systolic pressure (RVSP) ≥ 70 mmHg] (*n* = 8); (4) ASD closure with fenestration type (*n* = 6); and (5) absence of echocardiography between 6 months and 1 year after ASD closure (*n* = 78). Finally, 91 (26.1%) patients with significant TR (at least moderate degree) before ASD closure were included (Figure 1). Patients' clinical data recorded before ASD closure were obtained from hospital records. The study protocol was developed according to the principles of the Declaration of Helsinki and was approved by the Institutional Review Board of Severance Hospital.

### Echocardiography

Transthoracic echocardiography was performed using a standard ultrasound machine (Vivid E9; GE Medical Systems; Wauwatosa, WI, Philips iE33; Philips Healthcare; Netherlands) with a 2.5–3.5 MHz probe. Standard echocardiographic measurements were performed according to the recommendations from the American Society of Echocardiography guidelines (11). Assessment of the right side of the heart included the measurements of RV basal and mid-cavity at the end-diastole and end-systolic and diastolic area of the right ventricle on a four-chamber view. Dimension of the tricuspid annulus was measured at end-diastole on an RV-focused four-chamber view (11). For a comprehensive assessment of RV systolic function, RV end-systolic and end-diastolic areas were traced in a focused RV apical view, and RV fractional area change (FAC) was calculated using the following formula: FAC = [(diastolic area–systolic area)/diastolic area] × 100%. Tissue Doppler tricuspid lateral annular peak systolic velocity (S') was measured from the RV-focused apical view. The severity of TR was evaluated using multiple parameters (12). TR jet area was measured at the time of mid-systole in the apical four-chamber view using the area trace method. The vena contracta width of TR was measured at its narrowest point as it passes through the orifice. Estimated PA systolic pressure (PASP) was calculated from peak TV flow velocity using the modified Bernoulli equation, and right atrial pressure was estimated using the respiratory index of the inferior vena cava (13).

### Speckle tracking echocardiography

Speckle tracking echocardiography was performed by an experienced cardiologist blinded to clinical data, using a vendor-independent software package (TomTec software; Image Arena 4.6, Munich, Germany). All echocardiograms were uploaded in the Digital Imaging and Communications in Medicine format to the software package. For myocardial deformation analysis, the endocardial border was traced on the end-systolic frame in each selected image. The end-systolic frame (≥ 50 frames per second) was defined by the QRS complex or based on the smallest ventricular volume during a cardiac cycle. The software automatically tracked speckles along the endocardial border and myocardium throughout the cardiac cycle. The myocardium of the right ventricle was divided into six segments (basal, mid, and apical segments) of the RV free wall and septum. For the assessment of RV strain, we evaluated the average value of the longitudinal peak systolic strain from all segments of the free and septal walls of the right ventricle (RV GLS) in the RV-focused apical view. For the assessment of left ventricular global longitudinal strain (LV GLS), the value for LV GLS was obtained by averaging all segmental strain values from the 18 LV segments in the apical four-, three-, and two-chamber views. The absolute value of RV GLS and LV GLS was expressed as |RV GLS| and |LV GLS|, respectively.

### Assessment of RV–PA coupling

RV–PA coupling was estimated non-invasively using the conventional echocardiographic parameters and RV strain. Conceptually, RV–PA uncoupling occurs when RV contractility cannot rise further to match RV afterload (9–11). RV contractility was assessed by RV FAC, TV annular S', and |RV GLS|. Furthermore, PASP was used as a parameter of RV afterload. Finally, RV–PA coupling parameters were derived using the formula: RV FAC/PASP ratio, TV annular S'/PASP ratio, and |RV GLS| /PASP ratio.

### Follow-up

Echocardiography was routinely performed at 6 months to 1 year after ASD closure. Persistent TR was defined as a significant TR (at least moderate degree) even after ASD correction on echocardiography. Clinical outcomes were defined as a composite of cardiovascular death, HF admission, urgent hospital visits due to HF aggravation, and up-titrated diuretics.

### Statistical analysis

Continuous variables are presented as a mean ± standard deviation (SD) or median (interquartile range) and were compared using paired Student's *t*-test (for normally distributed data) or the Mann–Whitney U-test (for non-normally distributed data). Categorical variables are presented as absolute numbers and percentages and were analyzed using the chi-square or Fisher's exact test. Kaplan-Meier survival analyses and log-rank tests were used to compare the clinical outcomes between patients with persistent TR and those with resolved TR during the follow-up period. The cut-off values for parameters were determined as the values that maximized the sum of the sensitivity and specificity for persistent TR after ASD closure in the receiver operating characteristic (ROC) curve analysis. Logistic regression analysis was performed to determine the relationships between clinical and echocardiographic variables and persistent TR after ASD closure. The variables selected for entry into the multivariate analyses were those with a *p* < 0.10 in the logistic univariate analysis. The multivariate analyses focusing on RV function were performed in model 1. In model 2, the analyses including RV–PA coupling parameters were performed. A two-sided *p* < 0.05 was considered statistically significant. All analyses were performed using IBM SPSS Statistics for Windows, version 25.0 (IBM Corp., Armonk NY, USA).

## Results

### Baseline characteristics

**Figure 2:**
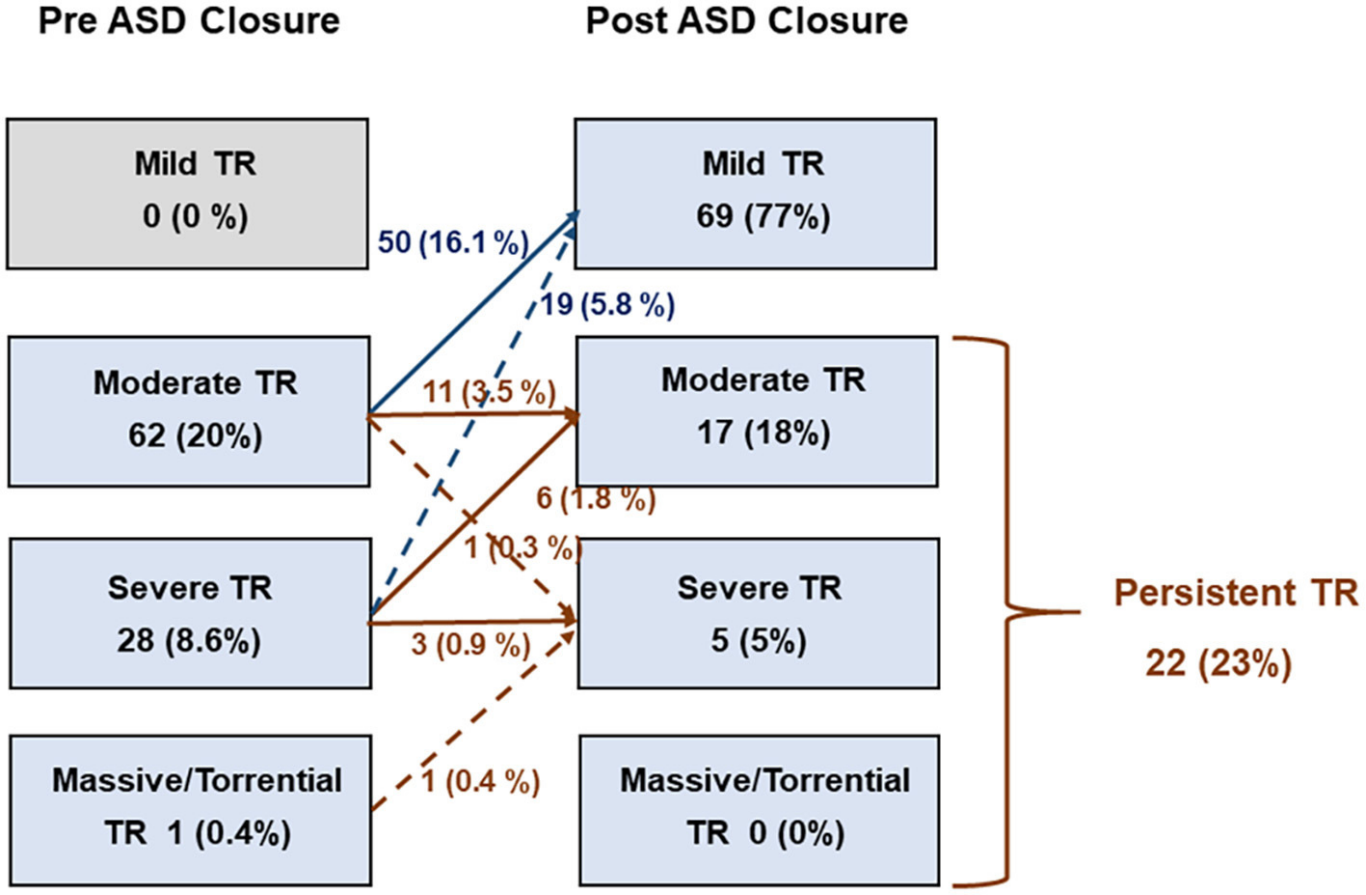
Changes in tricuspid regurgitation after closure of atrial septal defect.

The baseline characteristics of the study population, according to the presence of persistent TR, are presented in Table 1. The mean age was 55 ± 14 years and 62 (68.1%) patients were women. The average size of ASD was 2.43 ± 0.87 mm and the mean pulmonary-systemic flow ratio (Qp/Qs) was 2.44 ± 0.81. In total, 56 (61.5%) patients in our study population underwent percutaneous ASD closure and 35 (38.5%) patients underwent surgical ASD closure. Echocardiography performed at 6 months to 1 year after ASD closure revealed 22 (24.1%) patients with persistent TR (Figure 2). Patients who had persistent TR were significantly older (66 ± 10 vs. 52 ± 14 years, *p* < 0.001), had more hypertension (50.0 vs. 14.4%, *p* = 0.040), and had a higher prevalence of atrial fibrillation (AF) (50.0 vs. 23.1%, *p* = 0.008) compared to patients with resolved TR. There were no significant differences in ASD size, Qp/Qs, and type of intervention between patients with persistent TR and those with resolved TR.

**Table 1:** Baseline characteristics of the study population.

	**Total (*n* = 91)**	**Resolved TR (*n* = 69)**	**Persistent TR (*n* = 22)**	***P*** **-value**
Age, years	55 ± 14	52 ± 14	66 ± 10	<0.001
Female sex, *n* (%)	62 (68.1)	49 (71.0)	13 (59.0)	0.168
Body mass index, kg/m^2^	23.6 ± 4.3	23.4 ± 2.6	23.7 ± 3.3	0.445
Hypertension, *n* (%)	25 (27.5)	14 (14.4)	11 (50.0)	0.040
Diabetes mellitus, *n* (%)	8 (8.8)	5 (7.2)	3 (13.6)	0.234
Atrial fibrillation, *n* (%)	27 (29.7)	16 (23.1)	11 (50.0)	0.008
Systolic BP, mmHg	122 ± 18	123 ± 16	121 ± 17	0.353
Diastolic BP, mmHg	75 ± 13	75 ± 15	76 ± 11	0.490
Heart rate, bpm	71 ±14	71 ± 13	73 ± 13	0.432
ASD size, cm	2.43 ± 0.87	2.44 ± 0.72	2.43 ± 0.93	0.236
Qp/Qs ratio	2.44 ± 0.81	2.46 ± 1.82	2.24 ± 0.79	0.597
Type of closure, *n* (%)				
Percutaneous	56 (61.5)	39 (56.5)	17 (77.2)	
				0.461
Surgical	35 (38.5)	30 (43.4)	5 (22.7)	
Type of ASD, *n* (%)				
Secondum ASD	82 (90.1)	65 (94.2)	17 (77.1)	
				0.078
Primum ASD	9 (9.8)	4 (5.8)	5 (22.7)	

TR, tricuspid regurgitation; BP, blood pressure; ASD, atrial septal defect; QP/Qs; pulmonary blood flow/systemic blood flow.

### Cardiac remodeling and change in TR after ASD closure

Transthoracic echocardiography was performed at a median of 195 days (range 174–245 days) after ASD closure. The echocardiographic parameters were significantly changed after ASD closure (Table 2). Compared with baseline echocardiography, there was a significant reduction in the RV and right atrial (RA) sizes by reducing RV volume overload, along with an increase in the two-dimensional diameters of LV. In the RV systolic functional parameters, RV FAC decreased after ASD closure. Additionally, Tricuspid annular S' as an indicator of RV longitudinal function decreased significantly, but these parameters were within the normal range. There was a significant reduction in TR severity. Several parameters evaluating the TR severity, such as TR jet area, TR vena contracta, and end-diastolic TV annulus diameter, were significantly reduced after ASD closure. Furthermore, PASP decreased with a reduction in peak TR velocity.

**Table 2:** Echocardiographic parameters before and after ASD closure.

	**Pre ASD closure**	**Post ASD closure**	***P*** **-value**
**Left chamber parameters**			
LVEDD, mm	40.4 ± 6.0	47.0 ± 4.2	<0.001
LVESD, mm	26.8 ± 5.0	30.2 ± 4.2	<0.001
LVEF, %	62.8 ± 9.0	66.9 ± 7.3	<0.001
LA volume index, ml/m^2^	40.2 ± 15	40.5 ± 16	0.848
E velocity, m/s	0.78 ± 0.21	0.77 ± 0.24	0.719
A velocity, m/s	0.66 ± 0.17	0.62 ± 0.16	0.046
e' velocity, cm/s	0.76 ± 0.22	0.79 ± 0.17	0.499
E/e'	10.3 ± 3.0	11.6 ± 4.5	0.007
**Right chamber parameters**			
PASP, mmHg	45.7 ± 9.0	30.3 ± 8.1	<0.001
RV end-systolic area, cm^2^	18.8 ± 4.9	13.2 ± 3.9	<0.001
RV end-diastolic area, cm^2^	31.2 ± 7.2	21.2 ± 5.6	<0.001
RV basal diameter, mm	50.0 ± 6.3	40.9 ± 6.2	< 0.001
RV mid diameter, mm	42.9 ± 6.6	31.4 ± 5.6	<0.001
RA area, cm^2^	26.8 ± 7.2	17.5 ± 5.1	<0.001
RA major dimension, mm	63.1 ± 8.7	52.2 ± 8.8	<0.001
RA minor dimension, mm	46.5 ± 8.2	36.7 ± 5.8	<0.001
Tricuspid annular S', cm/s	13.9 ± 4.5	10.9 ± 2.8	<0.001
RV FAC, %	40.0 ± 8.2	38.1 ± 7.2	0.040
**Tricuspid valve parameters**			
Significant TR, n (%)	91 (100)	22 (24.1)	
TR jet area, cm^2^	6.4 ± 4.2	2.5 ± 3.0	<0.001
TR vena contracta, mm	5.7 ± 1.5	2.9 ± 2.3	<0.001
TR velocity, m/sec	3.0 ± 0.4	2.4 ± 0.4	<0.001
TV annulus diameter, mm	40.6 ± 4.1	32.1 ± 6.8	<0.001

ASD, atrial septal defect; LVEDD, left ventricular end-diastolic dimension; LVESD, left ventricular end-systolic dimension; LVEF, left ventricular ejection fraction; LA, left atrial; E, early diastolic mitral inflow; A, late diastolic mitral inflow; e', early diastolic mitral annular tissue Doppler; E/e', ratio of early diastolic mitral inflow to early diastolic mitral annular tissue Doppler velocity; PASP, pulmonary arterial systolic pressure; RV, right ventricle; RA, right atrium; FAC, fractional area change; TR, tricuspid regurgitation.

### Comparison of echocardiographic parameters between patients with resolved TR and those with persistent TR

Echocardiographic parameters of the resolved TR and persistent TR groups after ASD closure are described in Table 3. LV chamber size and *E*/*e*' were not different between the two groups. Right cardiac chamber size was similar between the two groups. In RV functional parameters, TV annular S' and |RV GLS| were significantly lower in patients with persistent TR than in those with resolved TR, but RV FAC was not different. In terms of RV–PA coupling, both TV annular S'/PASP ratio and |RV GLS|/PASP ratio illustrated significantly lower values in patients with persistent TR compared with those with resolved TR. TR jet area was significantly larger in patients with persistent TR, but other TV parameters were not significantly different between the two groups.

**Table 3:** Comparison of two groups based on the resolution or persistence of TR.

	**Resolved TR (*n* = 69)**	**Persistent TR (*n* = 22)**	***P*** **-value**
**Left chamber parameters**			
LVEDD, mm	40.9 ± 6.0	41.6 ± 5.8	0.643
LVESD, mm	27.2 ± 4.6	27.8 ± 4.9	0.581
LVEF, %	62.6 ± 9.1	63.6 ± 8.5	0.581
|LV GLS|, %	20.24 ± 3.43	19.61 ± 5.17	0.631
E velocity, m/s	0.79 ± 0.22	0.76 ± 0.17	0.571
E/e'	10.1 ± 3.1	11.3 ± 2.6	0.142
**Right chamber parameters**			
PASP, mmHg	45.7 ± 9.9	49.1 ± 8.8	0.157
RV End-systolic area, cm^2^	19.1 ± 4.9	18.2 ± 4.5	0.469
RV End-diastolic area, cm^2^	32.2 ± 7.9	30.1 ± 7.1	0.307
RA area, cm^2^	26.2 ± 7.3	29.1 ± 6.0	0.118
RV FAC, %	39.8 ± 8.2	39.1 ± 6.4	0.732
Tricuspid annular S', cm/s	13.4 ± 4.6	11.2 ± 3.0	0.042
|RV GLS|, %	20.6 ± 4.8	17.6 ± 6.4	0.032
RV FAC/ PASP, %/mmHg	0.86 ± 0.33	0.83 ± 0.21	0.623
TV annulus S' / PASP, cm/s*mmHg	0.31 ± 0.10	0.24 ± 0.08	0.011
|RV GLS| / PASP ratio, %/mmHg	0.46 ± 0.14	0.37 ± 0.13	0.013
**Tricuspid valve parameters**			
TR jet area, cm^2^	5.9 ± 3.6	8.1 ± 5.6	0.047
TR vena contracta, mm	5.7 ± 1.5	5.9 ± 1.5	0.457
TR velocity, m/sec	3.0 ± 0.3	3.1 ± 0.4	0.356
TV annulus diameter, mm	40.2 ± 4.0	41.8 ± 4.2	0.116

GLS, global longitudinal strain; ASD, atrial septal defect; LVEDD, left ventricular end-diastolic dimension; LVESD, left ventricular end-systolic dimension; LVEF, left ventricular ejection fraction; LA, left atrial; E, early diastolic mitral inflow; A, late diastolic mitral inflow; e', early diastolic mitral annular tissue Doppler; E/e', ratio of early diastolic mitral inflow to early diastolic mitral annular tissue Doppler velocity; PASP, pulmonary arterial systolic pressure; RV, right ventricle; RA, right atrium; FAC, fractional area change; TR, tricuspid regurgitation.

### Clinical outcomes between patients with resolved TR and those with persistent TR

During the follow-up duration (median: 22 months), there were no cardiovascular death and HF admission in patients with resolved TR and those with persistent TR. One (1.4%) of the 69 patients with resolved TR and 2 (9.0%) of the 22 patients with persistent TR were urgent visits due to HF aggravation. The diuretics dose was up-titrated in four (18.1%) patients with persistent TR and one (1.4%) patient with resolved TR. There was no significant difference in diuretics (furosemide) dose in patients with resolved TR and persistent TR (32 vs. 35 mg, *p* = 0.116). During follow-up, the diuretics dose was significantly higher in patients with persistent TR compared to the patients with resolved TR (34 vs. 20 mg, *p* = 0.023). Kaplan-Meier analysis showed that the event-free survival rate was worse in patients with persistent TR than in those with resolved TR (log-rank test; *p* < 0.001) (Supplementary Figure 1).

### Associating factors with persistent TR

Persistent TR after ASD closure was observed in 22 (24.1%), including 17 (19%) with moderate TR and 5 (5%) with severe TR (Figure 2). In univariate logistic regression analysis, age, hypertension, AF, TV annulus diameter, TR jet area, TV annulus S', |RV GLS|, and RV–PA coupling parameters were significantly associated with persistent TR after ASD closure. A multivariate logistic regression analysis was performed in two models. In model 1, only age remained an independent predictor for persistent TR after ASD closure. In model 2, age and |RV GLS|/PASP as an RV–PA coupling index were significant factors for persistent TR after ASD closure (Table 4).

**Table 4:** Pre-procedural factors associated with persistent TR after ASD closure.

	**Univariate analysis**		**Multivariate analysis**			
			**Model 1: |RV GLS|**		**Model 2: |RV GLS| /PASP**	
	**Odds ratio (95% CI)**	***P*** **-value**	**Odds ratio (95% CI)**	***P*** **-value**	**Odds ratio (95% CI)**	***P*** **-value**
Age	1.099 (1.045–1.157)	<0.001	1.075 (1.010–1.144)	0.022	1.071 (1.007–1.140)	0.030
Female sex	0.456 (0.169–1.229)	0.121				
HTN	5.169 (1.839–14.532)	0.002	2.595 (0.683–9.865)	0.162	2.594 (0.645–10.434)	0.179
DM	3.611 (0.821–15.879)	0.089	0.739 (0.126–4.346)	0.738	0.537 (0.087–3.304)	0.502
Atrial fibrillation	3.312 (1.212–9.054)	0.020	0.503 (0.126–4.346)	0.410	0.554 (0.121–2.540)	0.447
ASD size	0.715 (0.436–1.176)	0.186				
Qp/Qs ratio	1.202 (0.816–1.771)	0.353				
TV annulus diameter	0.998 (0.952–1.046)	0.943				
TR jet area	1.123 (0.003–1.257)	0.043	1.152 (0.981–1.362)	0.084	1.133 (0.961–1.336)	0.136
TR vena contracta	1.204 (0.919–1.578)	0.178				
PASP	1.038 (0.988–1.091)	0.136				
LVEF	1.019 (0.961–1.079)	0.534				
E/e'	1.080 (0.943–1.237)	0.267				
|LV GLS|	1.007 (0.947–1.072)	0.815				
Tricuspid annular S'	0.840 (0.711–0.993)	0.041				
RV FAC	1.004 (0.957–1.054)	0.870				
|RV GLS|	0.853 (0.766–0.950)	0.004	0.877 (0.760–1.012)	0.072		
Tricuspid annular S' /PASP	0.000 (0.000–0.0124)	0.009				
RV FAC/PASP	0.337 (0.072–1,566)	0.165				
|RV GLS| /PASP	0.000 (0.000–0.018)	0.001			0.001 (0.000–0.175)	0.012

### Associations between RV–PA coupling and persistent TR after ASD closure

**Figure 3:**
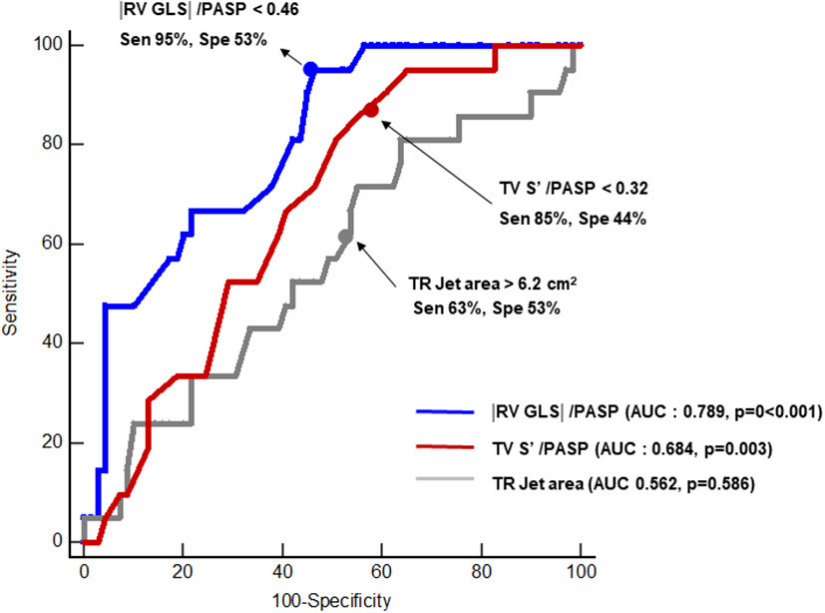
Receiver operating characteristic analysis comparing predictive values of tricuspid annular S' /PASP, |RV GLS|/PASP and TR jet area for predicting persistent TR after closure of ASD. AUC, area under the curve; Sen, sensitivity; Spe, specificity; RV GLS, right ventricular global longitudinal strain; PASP, pulmonary arterial systolic pressure; TR, tricuspid regurgitation.

**Figure 4:**
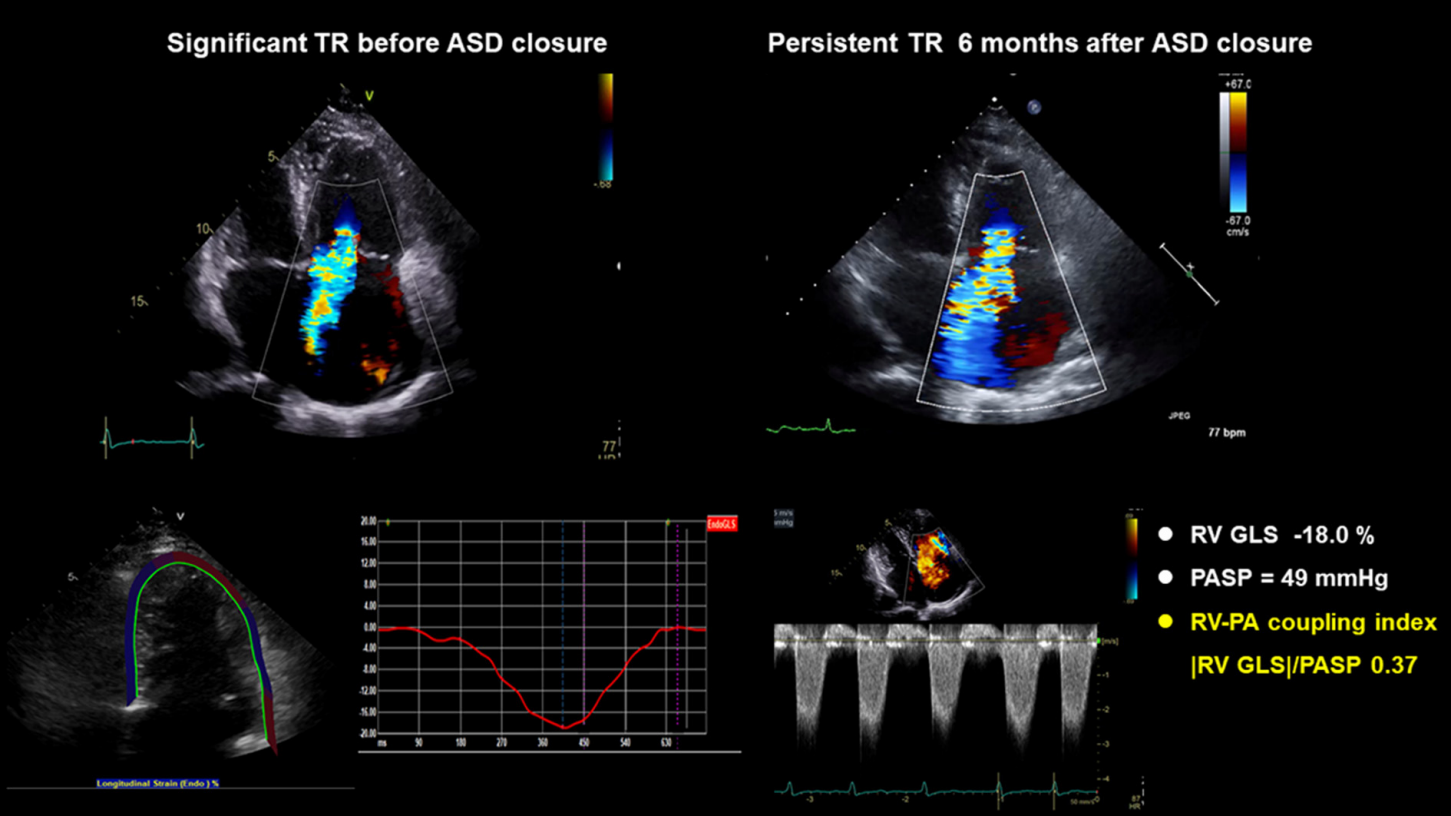
A representative case: A 72-year-old woman with hypertension, diabetes, atrial fibrillation, and |RV GLS|/PASP ratio before ASD closure < 0.46, had persistent TR after ASD closure. ASD, atrial septal defect; RV GLS, right ventricle global longitudinal strain; PASP, pulmonary arterial systolic pressure; TR, tricuspid regurgitation.

ROC was performed to evaluate the associations between RV–PA coupling and persistent TR after ASD closure. Parameters of RV–PA coupling demonstrated good predictive value (Tricuspid annular S' /PASP: area under the curve = 0.684, *p* = 0.003; cut-off value: 0.32; |RV GLS|/PASP: area under the curve = 0.789; *p* < 0.003, cut-off value: 0.46) (Figure 3). In a comparison of ROC in the RV–PA coupling index, the area under the ROC curve value of |RV GLS|/PASP was statistically significantly higher than that of tricuspid annular S' /PASP (*p* < 0.012). |RV GLS| was also a good cut-off value, but the area under the ROC curve value of |RV GLS|/PASP was statistically significantly higher than that of |RV GLS| [area under the curve: 0.729 (0.607–0.81)] (*p*-value: 0.043) (Supplementary Figure 2). TR jet area was not a significant factor in predicting persistent TR after ASD closure. Figure 4 displays a representative case of persistent TR after ASD closure, which showed that |RV GLS|/PASP ratio before ASD closure was lower than 0.46.

## Discussion

The principal findings of this study are as follows: (1) Persistent TR after ASD closure is not uncommon; (2) Old age and abnormal RV–PA coupling are associated with persistent TR after ASD closure; (3) Among the RV–PA coupling parameters, |RV GLS|/PASP < 0.46 depicts the most satisfactory predictive performance for persistent TR after ASD closure.

There is a growing interest in functional TR, which is associated with poor prognosis (4, 14). Dilatation of the tricuspid annulus secondary to RV enlargement, RA enlargement, tethering of tricuspid leaflets, and papillary muscle displacement are the main mechanisms of functional TR. As functional TR frequently occurs in adult patients with ASD, a few studies were focused on the change in functional TR after the closure of ASD (2, 6, 15). Functional TR was ameliorated after percutaneous and isolated surgical ASD closures, although half of the persistent TR cases remained in short-term follow-ups (2). Furthermore, pulmonary hypertension before ASD closure was associated with persistent TR after ASD closure (2). In a long-term follow-up study, significant TR decreased during the long-term follow-up period after transcatheter ASD closure, along with an improvement in heart failure symptoms (6, 15). However, even in long-term follow-up studies, significant TR persisted in approximately 20% of patients (6, 15). In our study, we followed up for 6 months to 1 year after ASD closure (surgical or percutaneous). Similar to the findings of previous studies, in our study, significant TR persisted in 22% of the patients after ASD closure. In patients with persistent TR, the urgent visit due to HF aggravation and up-titrated diuretics were significantly higher than those with resolved TR in this study.

With regard to the factors associated with persistent TR after ASD closure, previous studies showed varied results (6, 15). The left-to-right shunt of ASD causes volume overload of the right atrium and right ventricle. Accompanied by the enlargement of the right heart, functional TR was influenced by the changes in the atrial and ventricular geometry and function (16). Thus, preprocedural factors of the right side of the heart affect persistent TR after ASD closure. In previous studies, baseline RA size was the only parameter associated with persistent TR after ASD closure (6). However, in our data, we found no differences in the RA and RV sizes between those with and without persistent TR after ASD closure. This may be because not only the geometry of the right side of the heart but also RV function and remodeling of TV can affect persistent TR after ASD closure. In other studies, the presence of pulmonary hypertension before ASD closure predicted persistent TR (2). Patients with ASD and left-to-right shunts had a risk of PA hypertension (17). Chronic exposure of the pulmonary vasculature to increased blood flow in patients with ASD may produce histological changes in the pulmonary arteries, causing luminal narrowing leading, to high pulmonary vascular resistance with pulmonary hypertension (18). Pulmonary hypertension in patients with ASD influences outcomes and is associated with increased morbidity and mortality (19, 20). In our study, PASP before ASD closure was not associated with persistent TR after ASD closure. This may be because of the 6-month to 1-year follow-up after ASD closure in our study, instead of the short-term follow-up of previous studies (15). Consistent with other studies with long-term follow-up after ASD closure, in this study, the PASP showed normalization during the follow-up period.

Interestingly, in this study, RV–PA coupling parameters were independent factors of persistent TR after ASD closure. Chronic volume overload of the right side of the heart leads to dilatation of the right atrium and right ventricle and effects such as right heart failure and pulmonary hypertension (21). Furthermore, secondary TR imposes a chronic volume overload on the right ventricle that can increase RV wall tension leading to myocardial fibrosis and changed RV geometry, directly contributing to impaired RV contractility (22).

RV remodeling is frequently associated with secondary TR, which may accelerate RV–PA uncoupling (23). RV–PA coupling is a comprehensive parameter for both RV contractility and RV afterload (9, 24). Noninvasively measured RV–PA coupling, using a ratio of RV systolic function and RV afterload, is of superior prognostic value compared with RV systolic function and has proven clinical implications in acquired heart disease and PA hypertension (9, 25). Recently, in the field of congenital heart disease, abnormal RV–PA coupling in chronic PR, even in the setting of normal RV ejection fraction, was correlated with exercise capacity. In the present study, we used non-invasively measured TV annular S'/PASP and RV GLS/PASP as a marker of RV–PA coupling. A previous study demonstrated that TV annular S'/RVSP and RV free wall strain /RVSP as RV–PA coupling was used as parameters for predicting successful weaning from ECMO compared with conventional criteria in patients with refractory cardiogenic shock (26). In the present study, TV annular S'/RVSP <0.32 and |RV GLS|/PASP <0.46 exhibited a good predictive value of persistent TR after ASD closure. The hemodynamic changes in ASD patients can be considered a combination of RV myocardial damage and pulmonary vasculopathy. Thus, it is important to evaluate the comprehensive RV function and pulmonary circulation in patients with ASD as RV–PA coupling.

Moreover, in our study, the included patients were relatively old. Old age was a significant factor contributing to persistent TR after ASD closure. This may suggest that long-standing remodeling of the right heart and pulmonary vasculature affects RV myocardial function and pulmonary circulation. Therefore, it may be suggested that concurrent or subsequent TR interventions should be considered in elderly patients and patients with RV–PA uncoupling. TR interventions could reduce RV volume overload (27); therefore, could have a positive effect on RV–PA coupling in patients with ASD.

### Study limitation

There are several limitations to this study. First, this was a single-center, retrospective study with small sample size. Furthermore, the number of patients with persistent TR after ASD closure was small. Second, the severity of TR was evaluated using the semi-quantitative method in this study. Recent guidelines of TR showed that the grading of TR using quantitative methods, such as the PISA method or volumetric method, to evaluate the severity of TR. Third, we did not evaluate invasive hemodynamic data of RV–PA coupling. Thus, further validation of non-invasively measured RV–PA coupling parameters is needed. Last, TAPSE/PASP, the most validated parameter of RV–PA coupling, could not be analyzed due to small numbers (*n* = 52) in this study.

## Conclusion

Persistent TR after ASD closure is not uncommon. Old age and RV–PA uncoupling could be associated with persistent TR after ASD closure. These findings suggest that concomitant or subsequent TR treatment should be considered in older patients with abnormal RV–PA coupling.

## Synopsis

- Echocardiographic parameters of RV–PA coupling were reduced in patients with persistent TR after ASD closure.
- Old age and abnormal RV–PA coupling are associated with persistent TR after ASD closure.
- In elderly patients with abnormal RV–PA coupling, concomitant or subsequent TR intervention may be considered.

## Data availability statement

The raw data supporting the conclusions of this article will be made available by the authors, without undue reservation.

## Ethics statement

The studies involving human participants were reviewed and approved by Institutional Review Board of Severance Hospital. The Ethics Committee waived the requirement of written informed consent for participation.

## Author contributions

SHL and CS: planning, conducting the study, and drafting the manuscript. SHL, YS, JK, SaL, JS, IC, D-YK, G-RH, J-WH, and CS: collecting and interpreting data. YS and CS: guarantor of the article. All authors contributed to the article and approved the submitted version.

## Funding

This study was supported in part by a faculty research grant from Yonsei University College of Medicine (6-2021-0096).

## Conflict of interest

The authors declare that the research was conducted in the absence of any commercial or financial relationships that could be construed as a potential conflict of interest.

## Publisher's note

All claims expressed in this article are solely those of the authors and do not necessarily represent those of their affiliated organizations, or those of the publisher, the editors and the reviewers. Any product that may be evaluated in this article, or claim that may be made by its manufacturer, is not guaranteed or endorsed by the publisher.

## Supplementary material

Supplementary Figure 1 Kaplan-Meier survival curve according to presence of TR after the closure of ASD.

## Abbreviations

TR: Tricuspid regurgitation
ASD: Atrial septal defect
RV: Right ventricular
RA: Pulmonary arterial
GLS: Global longitudinal strain
PASP: Pulmonary artery systolic pressure.
