# Supplementary figures and images for: Clinical significance of right ventricular–pulmonary arterial coupling in patients with tricuspid regurgitation before closure of atrial septal defect
Source: Front Cardiovasc Med. 2022 Nov 14;9:896711. doi: 10.3389/fcvm.2022.896711 (PMC9702057; doi:10.3389/fcvm.2022.896711)

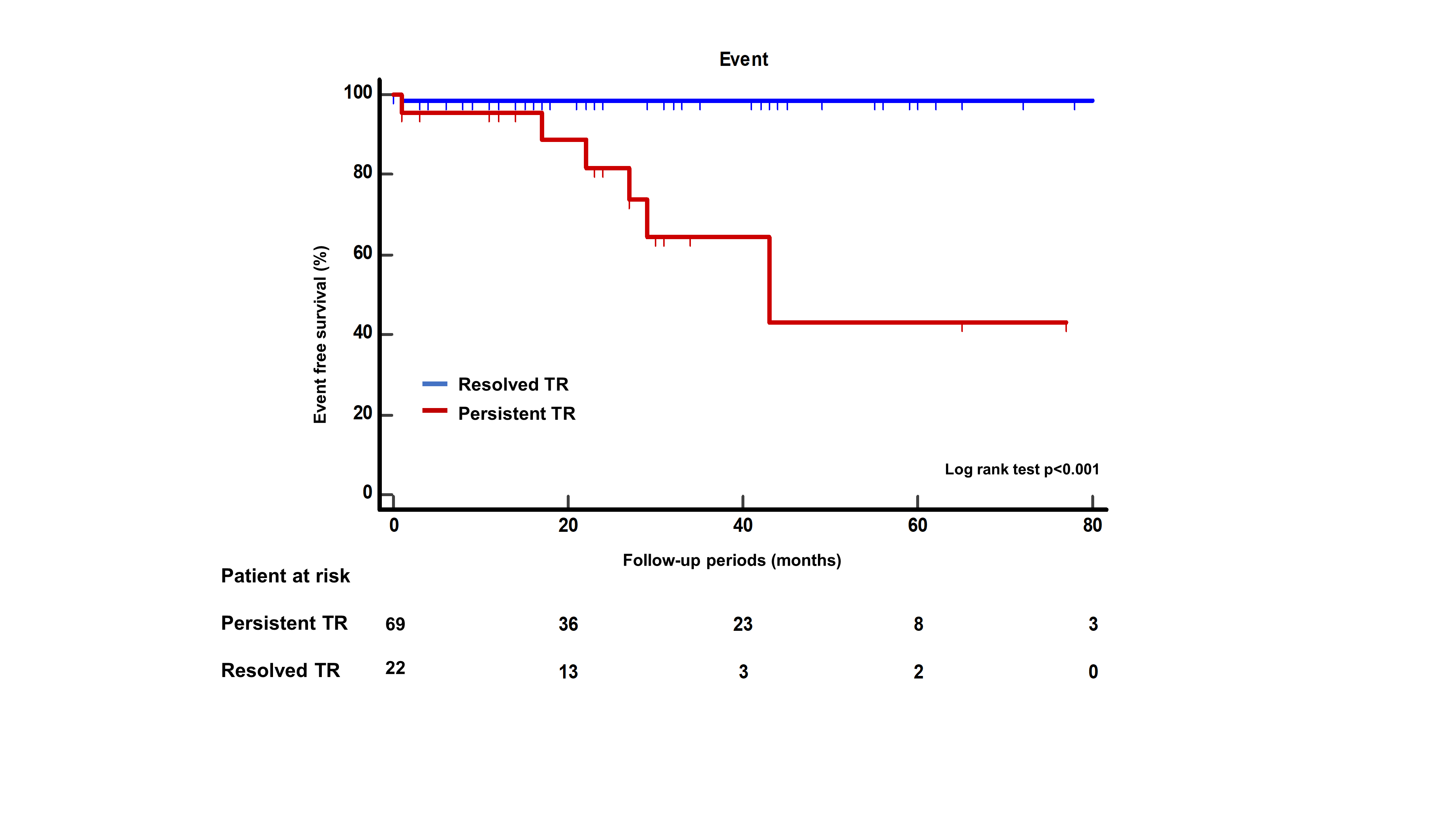

Supplement: Supplementary Figure 1 — Kaplan-Meier survival curve according to presence of TR after the closure of ASD. [file Image_1.TIF]

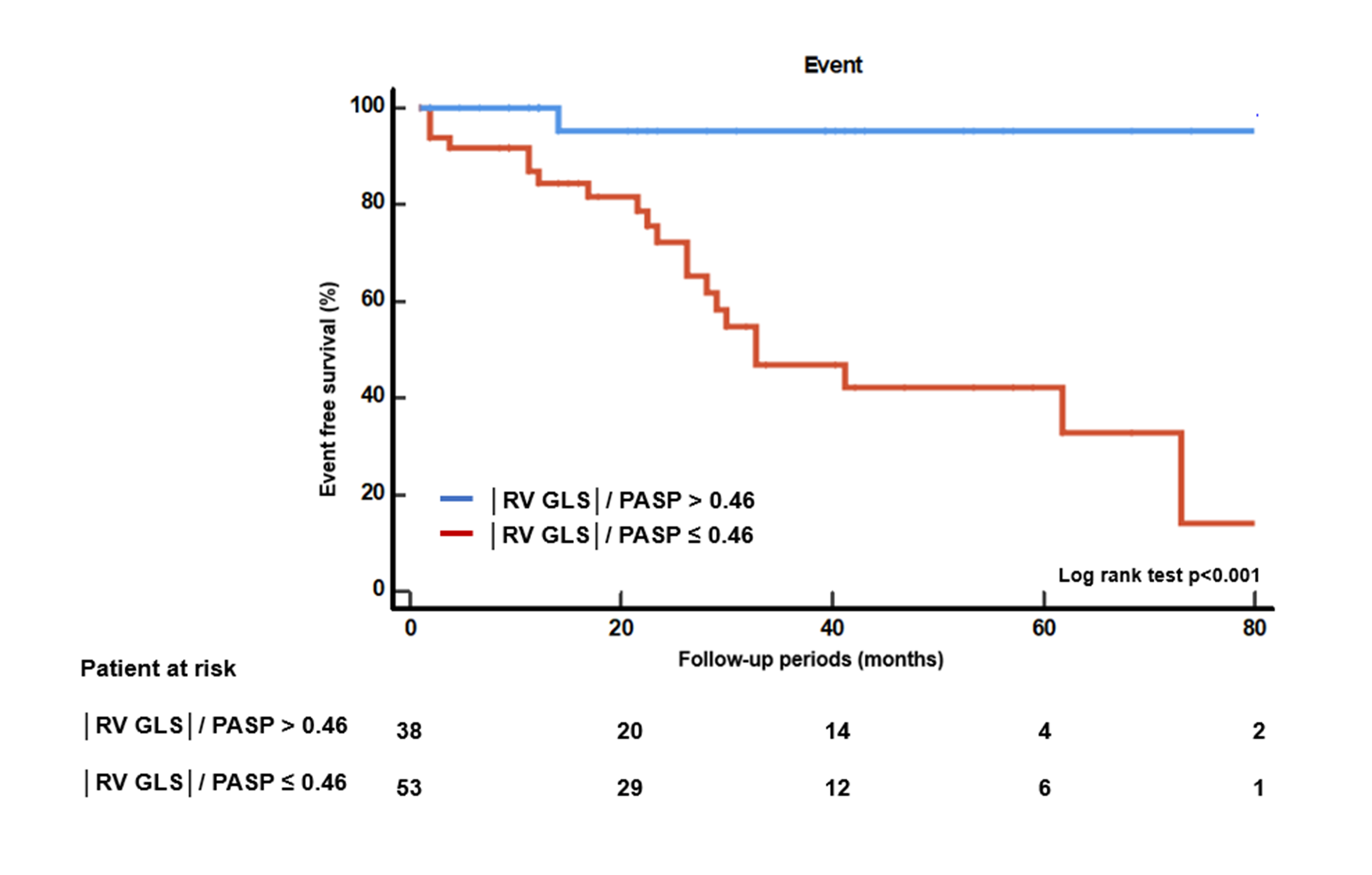

Supplement: Supplementary file 2 [file Image_2.TIF]
